# BRAIN - Holocene archaeo-data for assessing plant-cultural diversity in Italy and other Mediterranean regions

**DOI:** 10.1038/s41597-024-03346-5

**Published:** 2024-05-22

**Authors:** Anna Maria Mercuri, Eleonora Clò, Jessica Zappa, Giovanna Bosi, Elisa Furia, Cristina Ricucci, Matteo Di Lena, Federico Camerini, Assunta Florenzano

**Affiliations:** 1 Laboratorio di Palinologia e Paleobotanica, Dipartimento Scienze Vita, Università degli Studi di Modena e Reggio Emilia, Via Campi 287, 41125 Modena, Italy; 2 Doctorate School in Models and Methods for Material and Environmental Sciences, Dipartimento di Scienze Chimiche e Geologiche, Università degli Studi di Modena e Reggio Emilia, Via Campi 103, 41125 Modena, Italy; 3 Doctorate in Evolutionary Biology and Ecology, University of Rome Tor Vergata, Rome, Italy; 4 SIDEL S.p.a., Via La Spezia 241/A, 43126 Parma, Italy; 5 AD Consulting S.p.a., Via Pier Paolo Pasolini 15, 41123 Modena, Italy

**Keywords:** Biodiversity, Palaeoecology

## Abstract

In the field of botany applied to archaeological and palaeoecological studies, the multi- and inter-disciplinary nature of this research produces a lack of data sharing and scattered articles in the specialty literature or in national and international journals. The vast production of archaeobotany and palynology data makes it necessary to develop a tool for the availability, accessibility, and dissemination of existing research. Many databases exist on palaeoecology, archaeobotany or pollen data. There are no collections focused on archaeological sites and human-induced environments and centred on Southern Europe and the Mediterranean. BRAIN - Botanical Records of Archaeobotany Italian Network is the first database listing sites from which all types of plant records are available in Italy and nearby Mediterranean regions. BRAIN represents the largest integrated collection of archaeo/palaeo-botanical data and a range of descriptive information that makes data recovery FAIR ready. This unique network hosts data on the availability of anthropogenic pollen, palynomorphs and plant macroremains in the same database, and experts of different research fields may contribute to it.

## Background & Summary

### Synergy between botany and archaeology is key to understanding ongoing environmental transformations

The current environmental conditions are the result of the synergic action of climate and human agency. Much research on ecology and sustainable development focuses on the last few decades of Plastic Era, or at most on the important transformations that have taken place since the Industrial Revolution^1–5^. However, one has only to look at the archives unearthed by archaeology to realise that past cultures have deeply affected the forms of nature for millennia. Plants and waters, animals and rocks were the tools of cultural development before the explosion of fossil fuel technology, but the effects of this action are not as evident in the deep past as they are today. How long and continuous has our influence been on the different environments of the planet? How much is today’s landscape the result of its long history? By examining archaeological sites, that, by definition, are the places of anthropic presence, a sort of Pro-Anthropocene is clear and indisputable^6,7^.

The role of human impact in shaping prehistoric and historical landscapes has mainly been investigated as a topic specific to environmental archaeology^8,9^, also implying some review of regional demographic trends in archaeology (e.g.^10^). The combination of data coming from different historical, social and natural sciences in the study of archaeological sites, however, has fostered extremely rapid scientific development in the last decades. Botany has brought an innovative biological perspective to the archaeological approach as an elective tool for understanding the ecological dynamics that, with cultural pressures, led to the structure of modern vegetation^11,12^.

Moreover, what is the cultural dimension of our relationship with nature? When the observation of environmental transformations starts from prehistory, it is evident that cultural and environmental changes are strongly connected. Plants have been the first subsistence resource, providing food, fuel, and tools. By moving from dependence to the control of plant cycles, plants provided fields, and humans adapted their lifestyles to change. In this process, the human agency has left imprints with increasing evidence, from the invention of agriculture to the present land overuse. Much multidisciplinary research finds agreement that it is as early as the Neolithic period this influence has modified substantially in respect to previous phases^13^, but it is in the wood-based economy of the Bronze age civilizations that the process of exploiting natural resources was implanted and guided the following civilizations^14^.

Long-term complex dynamics underpin the agricultural vocation or the natural heritage of modern territories and cannot be ignored. This research deals with the cumulative pressure of humans on the environment that, even with backlashes, has been an especially critical issue in Europe and the Mediterranean during the Holocene^15,16^. In a broader sense, plants have played a central role in the economy and culture, and can now drive safeguard, conservation, renewal, and sustainable development in all countries.

### Archaeobotanical databases

The existing archaeological databases have often focused on collecting information from archaeological sites by focusing on the presence of macroscopic remains and on a limited chrono-cultural time span. This is determined by the fact that a database can have as its main purpose the collection of data from a single project, focused on a specific topic such as, for example, food resources during the Roman period, or on a specific type of record such as seeds/fruits in the Eastern Mediterranean (e.g., https://www.ademnes.de/). However, if the objective of research is to reconstruct environmental transformations on a long-term scale, it is also necessary to consider the anthropogenic impact on the territory, from a local to a broader scale, and its possible role in environmental changes. In this case, also the microscopic evidence should be analysed and sites within the area of influence of archaeological sites should be included in reconstruction studies. On the other hand, palaeoecological databases hardly include detailed research on archaeological sites, which are considered to be of very local relevance and not informative for regional reconstructions. The complexity of the transport of plant remains also complicates any interpretation of the integrated data: while humans are the main transport agents at an archaeological site, the source area can be wide, including travel and trade, or airborne arrival of pollen and seeds. Outside of an archaeological site, air or water transport can be prevalent, but it documents the joint effect of climate and humans on plant cover, with a range that is difficult to generalize as varies in each context.

This paper provides an overview of the status of BRAIN, where data from literature on plant micro- and macro- remains are assembled in the same database, and experts of different fields of research contributed to the collection of information. The database includes data from archaeobotanical research studies carried out in archaeological sites located in the Italian peninsula, Italian islands, and some other Mediterranean Sea countries. In this sense, BRAIN has a special application for the study of the plant uses and subsistence resources. This wide area has been constantly interested by variability across time and regional ecosystems resulting in complex landscapes, a mosaic of environments that have served as a stimulus and background for important cultural changes. The rhythms and characteristics of these transformations have varied and must be reconstructed considering the different geo-vegetational realities along coasts, plains and mountains in the Mediterranean countries.

Furthermore, as botanical data allow for cultural/environmental reconstructions from comparisons between different records in the same area, other human influenced sites located close (near- or off-) the archaeological sites, are added. Indeed, the inclusion of sites from outside Italy was not among BRAIN’s priorities and they were included at the authors’ request or in view of some synthesis publications that had included sites from different Mediterranean countries. In our knowledge, this is the largest database of this kind because the similar databases only include one type of chronology (e.g., Roman period) or one type of record (e.g., pollen), or include multidisciplinary records but none or few archaeological sites.

The detailed understanding of human-environment interactions needs the application of pollen analysis to the study of long-term human impact, the extent to which humans have acted on terrestrial and aquatic ecosystems, the onset of modern cultural landscapes and the eventual interference with climate change.

Food production and diet, the basis of any subsistence strategy, are investigated mainly through the macroremains, such as seeds/fruits of domestic crops, while charcoal/wood from useful trees and shrubs complement the data on fuel and resource availability. All studies are completed by molecular and isotopic data, statistical elaboration, and comparison with modern analogues. Results have often provided unknown palaeoethnobotanical aspects of the past human societies^17–19^. The modern research approach drives the exploration of new biological fields and requires deep inter- and intra-disciplinarity between different botanical fields. The BRAIN database aims to offer this tool to research in the field of archaeobotany to archaeologists and to palaeoecologists. It was designed with a dynamic structure, giving the possibility of adding fields and categories in the future, based on new scientific approaches and needs. Considering the existence of numerous other thematic databases in archaeology or botany, it might be very useful in the future to make links between this and other databases, in a kind of ‘network of networks’, rather than making redundant lists.

### BRAIN database on Italy’s archaeobotany as a research collection

In Italy, archaeobotany is at the forefront, and much research has been conducted that is rooted in the long history of human presence in this country. Besides the Pleistocene Paleolithic, countless sites have dotted the territory since the beginning of the Holocene. The extraordinary *repertoire* of archaeological and scientific data, often including botanical data, has been dispersed in many publications, often written in Italian and not always easily available.

Since 2014, as an output of the 9^th^ European Palynological Palaeobotanical Congress in Padua, palynologists and archaeobotanists started the recognition of all existing studies on plant records from Italian sites. Dealing with the Holocene and archaeobotany, the main output of the cooperative recognition was the scientific network BRAIN, proposed as an inventory of all existing plant data from archaeological sites in Italy^20^. A project of developing the initial activity in a true cooperative research action encouraged to present posters at congresses and to mention the network in several oral communications. The cooperative work of the contributors resulted in the further publication of joint papers^21,22^.

**Fig. 1:**
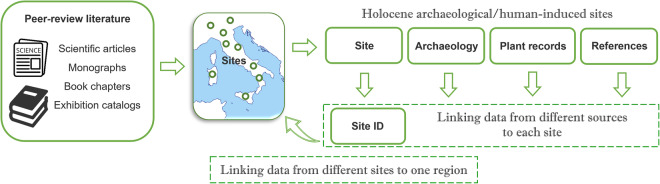
Archaeobotanical Data Collection, from peer-review literature to Site ID, for linking scattered data to one site, and many sites to one region/area.

Presented at the MedPalyno Congress of Rome in 2015 (currently the biannual meeting joining the Italian, Spanish and French palynologists), BRAIN is organised as a large collection of sites with metadata, available free online. Data were collected by compiling the geographical, chronological, archaeological, and scientific reference data of each site. Considering that the administrative management of the archaeological heritage in Italy is regionally distributed, the type of data collection allowed for a prompt location of sites in the relevant region. In Italy there are 20 regions, in the continental and insular land. The Republic of San Marino lying on the peninsula was added as a foreign country. Contexts and cultural chronology were then added, both updated with each new entry, as a main information useful for using the data. Further information collected was the type of plant record studied. In some cases, if research on different records, and in the framework of different projects, had been carried out by several groups, the analyses published at different sources were traced back to the same site, also linking scattered archaeobotanical data to one site, and many sites to one region/area (Fig. 1).

The use of the information for research purposes also required some implementations that were not immediately foreseen when BRAIN was first created. Over the past eight years, and behind continuous adjustments, BRAIN was implemented in several aspects to achieve the simplest use and maximum scientific utility achieved today:

A) new sites were added by the database curator (A.F.), whenever a new scientific article was published, even without a recommendation by the author(s);
B) the database has been extended to other contexts related to human presence, considering the following characteristics: B.1 ‘on-site’ are the archaeological sites, where human presence and action are obvious; B.2 ‘off-sites’ include terrestrial, lake and marine cores = to obtain a more complete picture of the research in a given territory, also contexts outside the narrow archaeological excavation but influenced by human presence, were included; human action in the Mediterranean has, in fact, been so extensive that it has left clear traces in the form of anthropogenic pollen indicators or fire growth, also discussed in research on sea cores^23,24^; B.3 ‘spot records’ refer to burials, pot content or other special contexts, obtained from one spot study, or material preserved in museums and not extracted from known stratigraphy;
C) the list of citations was filled providing the full reference list of papers with botanical analyses, sorted by alphabetical order of surname of the first author.

**Fig. 2:**
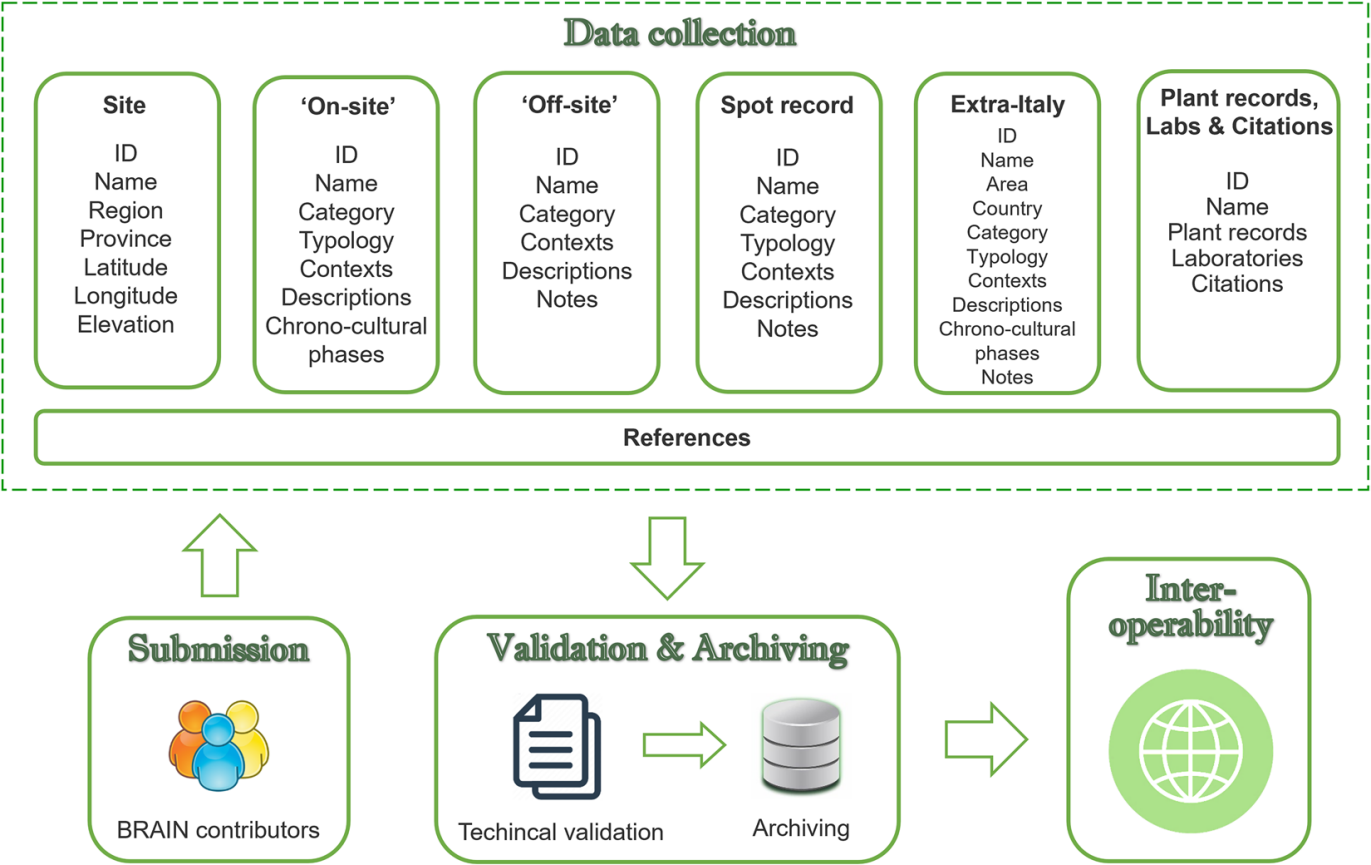
Overview of BRAIN workflow from metadata submission to online visibility.

The large accumulation of botanical data from past deposits makes it necessary to create this new dataset to share multi-proxy data distinguishing different site categories and providing a more accurate description of human-environment interactions^25,26^ (Fig. 2). The range of information obtainable is completed by reporting the presence of different types of plant records in the studies of one site. This is a fundamental step in reconnecting each piece of botanical evidence to its nature and assembling each plant part as a single organism.

## Methods

Created in 2015 and updated until 2023, the database aimed to fill a gap in the recognition of studied sites in Italy, published on peer-review and also on grey literature, sometimes not known and ignored by new studies. It includes metadata from sites that have been studied for archaeobotany and palynology, and reports what types of plant remains have been analysed. BRAIN offers to support the use of archaeobotanical data for scientific, educational, and disseminative purposes.

### Data collection

**Fig. 3:**
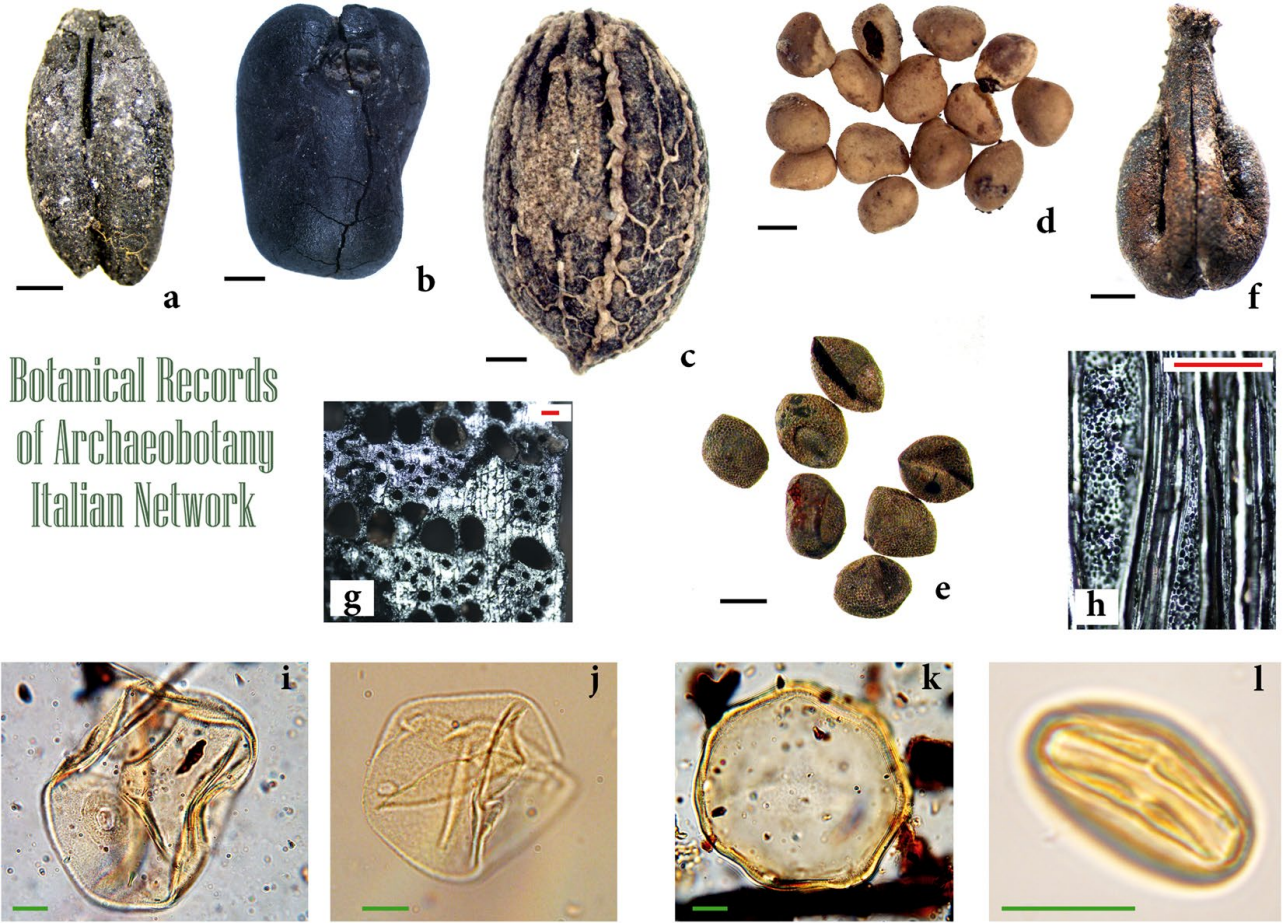
Archaeobotanical records and the ID of the archaeological site in BRAIN. (**a**) Caryopsis of *Hordeum vulgare* from NER29 (Pianella di Monte Savino); (**b**) Seed of *Vicia faba* var. *minor* from NER121 (Reggio Emilia, Piazza Vittoria); (**c**) Endocarp of *Olea europaea* from NSM1 (San Marino Republic, Domagnano); (**d**) Achenes of *Ficus carica* from NER80 (Parma, Piazza Garibaldi); (**e**) Seeds of *Brassica rapa* from NER34 (Ferrara, Corso Porta Reno/via Vaspergolo); (**g**) Charcoal of deciduous *Quercus* from NER73 (Montegibbio); (**h**) Charcoal of *Fagus* from NER73 (Montegibbio); (**i**) Pollen of *Avena/Triticum* group from CLA77 (Vulci); (**j**) Pollen of *Cannabis* from CLA77 (Vulci); (**k**) Pollen of *Juglans* from CLA77 (Vulci); (**l**) Pollen of *Castanea* from NER34 (Ferrara, Corso Porta Reno/via Vaspergolo). Photos by G. Bosi, R. Rinaldi, A. Benatti, M. Mazzanti. Scale bar: black = 1 mm; red = 100 µm; green = 10 µm.

The inventory of the sites was achieved through a combination of consulting the specialised literature and checking the completeness of the sources with the various researchers involved in archaeobotanical research. All available peer-reviewed literature reporting on archaeobotanical data was checked to verify which sites were studied and what kind of botanical remains were analysed. The work was conducted by consulting the known literature, online, in university libraries and citation databases (like SCOPUS), the book chapters on monographies and catalogues, and contacting the authors directly. Each site providing results on plant remains (Fig. 3), both microscopical (pollen, phytoliths, spores, cysts, other non-pollen remains, and starch) and macroscopical records (seeds, fruits, woods/charcoals, basketry, and all related hand-made records), even published in different papers and book chapters, has been entered into a dataset, under the same ID number (see below). Geographical and archaeological data have been added as reported in the scientific papers. Twelve chrono-cultural periods, from the Neolithic to the Modern age, have been identified within the Holocene.

### Reference list

For each site included in the database, the full literature list of the archaeobotanical studies includes articles in scientific journals, chapter in monographies, PhD thesis, congress abstracts, grey literature, published since the 1980ies. Papers dated before the ‘80ies were consulted only if they are key archaeobotanical publications for the site. Bibliographic citations for each site were associated with each site and reported in the database: they are essential for understanding the excavation context, the quantity of samples examined, and the techniques used to extract or isolate botanical records.

### ID criteria assignment

Each site included in the database was assigned a BRAIN ID code.

The ID of the sites located in Italy is composed by three parts (Table 1):

1. N = Northern, C = Central, S = Southern according to Italian geographical macroareas;
2. Two letters indicating the Italian administrative regions;
3. Consequential number, given automatically when entering a new site. As example: Terramare di Montale = located in northern Italy (N), in the Emilia Romagna region (ER), sixty-eighth site entry for the region = NER68.

**Table 1:** Macroareas and Regions in Italy as entered in BRAIN.

MACROAREA	REGION		LABEL
Northern = N	Emilia Romagna	ER	NER
	Friuli Venezia Giulia	FV	NFV
	Liguria	LI	NLI
	Lombardia (Lombardy)	LO	NLO
	Piemonte (Piedmont)	PI	NPI
	Trentino Alto Adige	TR	NTR
	Valle d’Aosta	VA	NVA
	Veneto	VE	NVE
Central = C	Abruzzo	AB	CAB
	Lazio (Latium)	LA	CLA
	Marche	MA	CMA
	Molise	MO	CMO
	Toscana (Tuscany)	TO	CTO
	Umbria	UM	CUM
Southern = S	Basilicata	BA	SBA
	Calabria	CL	SCL
	Campania	CA	SCA
	Puglia	PU	SPU
	Sardegna (Sardinia)	SA	SSA
	Sicilia (Sicily)	SI	SSI

The ID of sites located out of Italy is composed by two parts:

1. NSM is used for sites located in the Republic of San Marino; EXI refers to sites located in other countries outside Italy;
2. Consequential number, given automatically when entering a new site.

### Database: sites entry

The database includes a set of items that were filled at each new entry, as follows:

ID = ID number of the site in the BRAIN database

Name = name of the site

Region = Italian region or Country

Province = Italian administrative province

Latitude = geographical coordinates (decimal degrees)

Longitude = geographical coordinates (decimal degrees)

M a.s.l. = elevation above sea level

Category = on-site, off-site, spot record

Typology = settlement, cave/shelter, near/under sea site, sacred/funerary complex, monumental building (the archaeological typology may not correspond to the context from which the archaeobotanical data came)

Contexts = urban, rural, terramare, pile-dwelling, harbour, shipwreck, necropolis, tomb/burials, votive/ritual structure, mummies, tumulus, church, abbey, castle, sanctuary, temple

Descriptions = near site, domus, channel, silos-storage-granary, pit, latrine, productive area, hearths- oven, sewer, village, ditch

Plant records = pollen (p), non-pollen palynomorphs (npp), micro-charcoal particles (cp), phytoliths (ph), seed and fruit (S/F), wood (W), wood tool (Wt), charcoal (C), mould (M), textiles (T), basketry (Bk), adobe, bread or similar food, leaves and microsporophylls, mastic, moss, plant tissues, ropes, straw, wick (Ot).

Chrono-cultural phase = Mesolithic (M), Neolithic (N), Chalcolithic (Ch), Bronze age (B), Iron age (I), Etruscan-Archaic period (E-A), Hellenistic period (H), Roman age (R), Medieval ages (Ma), Renaissance (Re), Modern age (Mo).

Laboratories = Affiliation of the BRAIN contributors or Ot (independent researchers or laboratories not included in the BRAIN network)

References = citations

Notes = specific additional description

## Data Records

The site inventory includes 800 ID records collected from more than 980 research papers. The full dataset “Botanical Records of Archaeobotany Italian Network” is stored at Figshare^27^ and includes eight tables (spreadsheets) with the lists of sites and geographical data, type of site category, type of plant records and corresponding references. The individual tables are described below.

### TAB 1BRAIN: Sites with botanical records, ID and corresponding geographical metadata

**Fig. 4:**
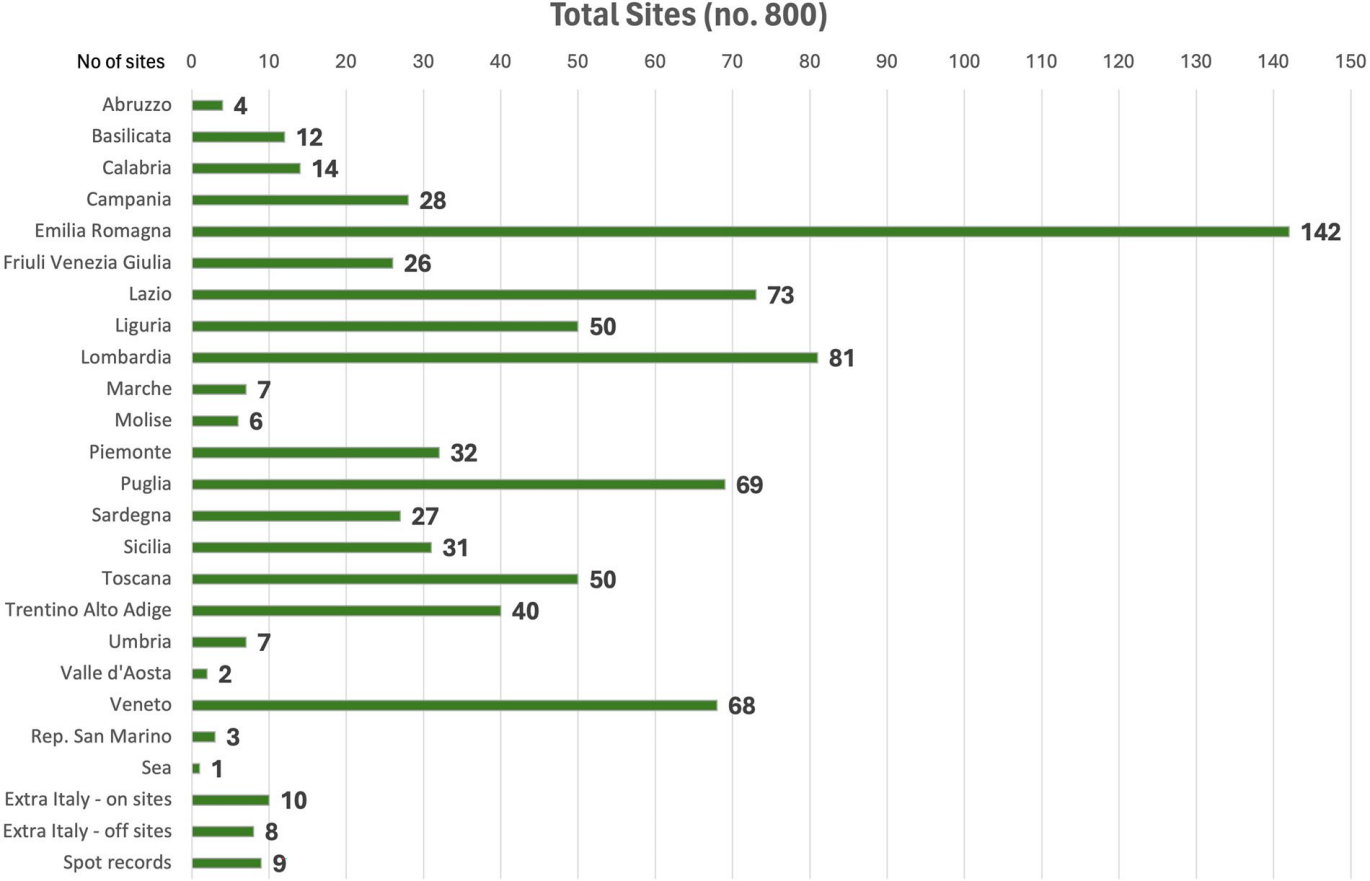
Total number of sites with archaeobotanical analyses in BRAIN, in the 20 Italian regions and outside Italy, as resulting from the comprehensive literature review on the subject and on the information gathered from consulting analysts.

This table lists all the sites in the database. They have been archaeobotanically studied. The focus is on Holocene sites distributed across an area covering continental Italy, the Italian peninsula (including the Republic of San Marino), the islands^20^ and some neighbouring countries (Fig. 4). This census is essential to understanding how extended this research is, how many analyses have been done, how many human-modified settlements (on-sites) are distributed near human-influenced records such as lake and other terrestrial sedimentary sequences (off-sites)^28^.

#### Metadata

The structure of the spreadsheet is alphabetically by Italian Region, from Abruzzo to Veneto, and then the sites from the Republic of San Marino, the Adriatic Sea and Extra-Italy countries are listed (column A). For each geographical division, sites are listed in order of ID entry. BRAIN ID (column B) = Each site has an Identifier code that is the combination of letters (identifying the geographical area and region) and the number of site entry.

NAME (column C) = Name of the site, as reported in research papers. In the case of similar but not perfectly matching names for the same site, the name most frequently mentioned in scientific publications was chosen. In the case of many sites studied within a large site (e.g., a city), the concept of ‘multipoint site’ was used^20^: in this way, the same site name is followed by point specifications for each specific site studied. For example, Pompeii is a multipoint site where the IDs range from SCA11 to SCA19, as different points have been studied within different projects, and by different archaeological direction and teams. Other examples of multipoint sites are cities of the Emilia Romagna region (Bologna, Modena, Ferrara), Liguria (Genoa), Latium (Rome) and Apulia (Lecce).

REGION (column D) = The geographical region according to Italian territorial policy.

PROVINCE (column E) = The administrative province according to Italian territorial policy.

LATITUDE (column F) - LONGITUDE (column G) = Geographical coordinates are basically those reported in the scientific publications where the archaeobotanical data are available. Coordinates have been tested on Google Earth to verify their correct positioning.

METERS ABOVE SEA LEVEL (column H) = Elevation of the sites as reported in the scientific publications where the archaeobotanical data are available.

### TAB 2 BRAIN: List of archaeological sites with contexts and chrono-cultural phases, in the Italian peninsula (‘on-site’ category and archaeology)

This census reports the ‘on-sites’ listed in the database and corresponds to the archaeological sites from which excavations and stratigraphic layers have yielded data useful for archaeobotanical studies. It includes 739 archaeological sites from the 20 Italian regions plus 3 archaeological sites located in the Republic of San Marino.

This type of research deals with the local pressure of humans on the local environment, into or close to an archaeological site where the human presence and activity has been archaeologically demonstrated. Combining humanistic and scientific knowledge in the study of archaeological sites allows a more and more detailed environmental reconstruction, knowledge on the development of agrarian landscapes and understanding of the role of humans in changing niches of the Mediterranean landscape.

#### Metadata

REGION (column A), BRAIN ID (column B) and NAME (column C) are reported as in Tab.1 BRAIN.

CATEGORY (column D) = It is specified that the site is on-site (archaeological excavation).

TYPOLOGY (column E) = The typology of archeological site is attributed according to the archaeological literature, type of excavation and archeological study. In a few cases, according to the complexity of archaeological evidence, one site may belong to more than one typology and its different parts were studied within different research project; in this case, there are as many typologies as there are different analyses performed on different parts of that site (e.g., in Velturno – Tanzgasse NTR12 and in Montegrotto – via Neroniana NVE10 other typologies were identified besides ‘settlement’).

CONTEXTS (column F) = The urban, agricultural or cultual contexts are established based on the archaeological research and literature, for each specific site. Also in this case, there are as many contexts as there are different analyses performed on different contexts of that site (e.g., in S. Giovanni Persiceto - Piazza del Popolo NER9, two different Tomb/Burials and Rural contexts were analysed).

DESCRIPTIONS (column G) = This is the description of the point of sampling of plant macro or microremains; in many cases it was not possible to find this information, so many rows report ‘NA’ (Not Available); the different points of sampling are described according to the archaeological reconstruction of each site.

**Fig. 5:**
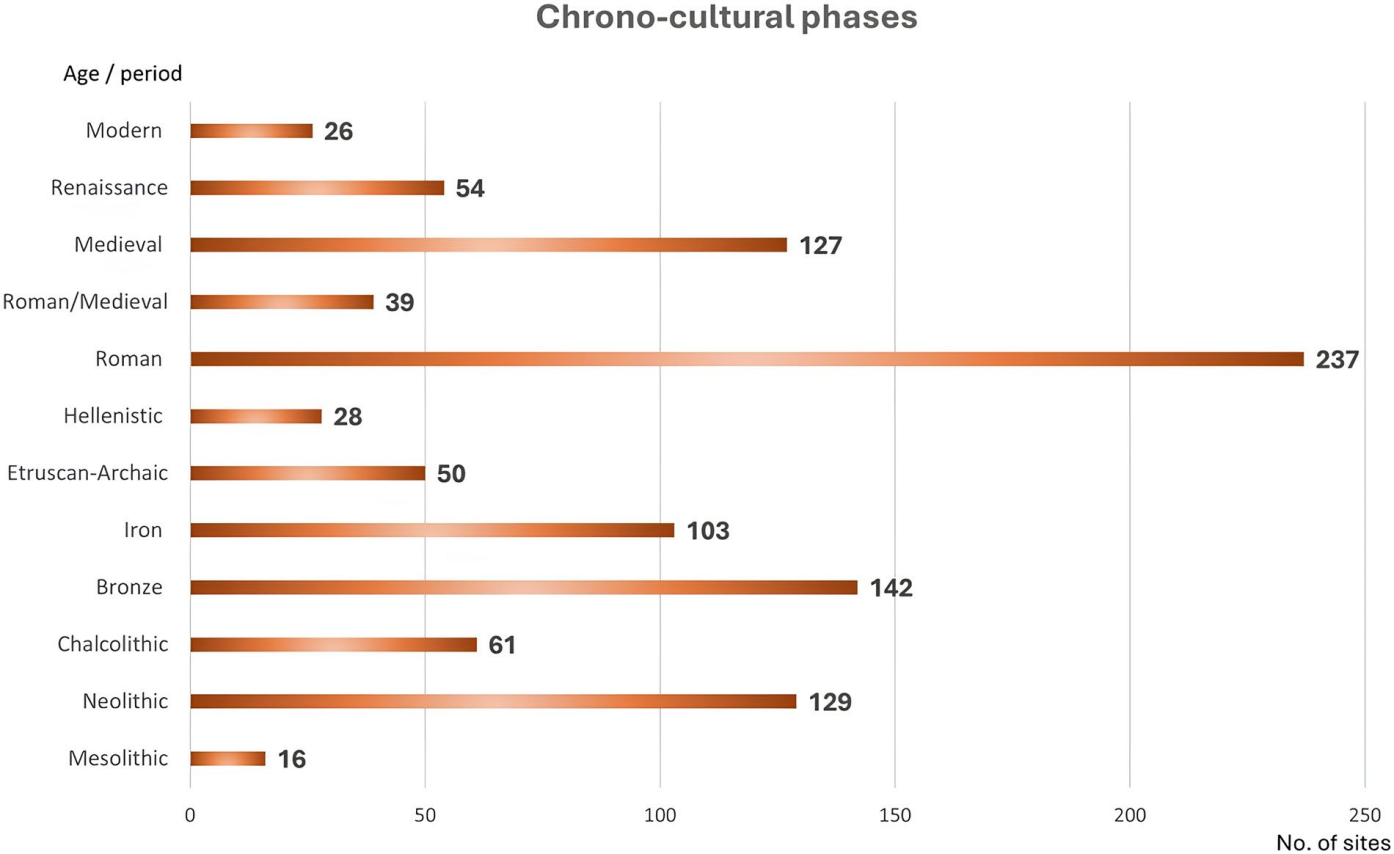
Total number of ‘on-sites’ with archaeobotanical analyses in BRAIN, in the main chrono-cultural phases of Italy and Mediterranean countries, as resulting from the overall review of the literature on the subject and based on the information provided by the analysts.

CHRONO-CULTURAL PHASES (column H) = The main cultures and chronological phases are reported as established in the archaeological literature, from the M = Mesolithic to the Mo = Modern age (Fig. 5). This is one of the most problematic points in archaeological research and sometimes presents many problems of attribution. In this data collection, every cultural/chronological attribution was based on the reference article. In the case of sites with more than one chronological phase, the provenance of the botanical samples was considered first, and in the case of several phases with archaeobotanical data, all chronological attributions of the archaeobotanical samples were indicated as reported in the published literature of the site. One site can cover different chronological-cultural phases and archaeobotanical samples therefore refer to different phases.

### TAB 3 BRAIN: List of other sites with contexts and notes, in Italy (‘off-site’ category)

As the research on environmental transformations deals with the cumulative pressure of humans on the environment, the interest of researchers is usually on a regional besides local reconstruction. Therefore, the comparison between on-site and off-site is very helpful to understand the deep nature of human-environment relationships. BRAIN includes 39 off-sites from marine, lake and terrestrial contexts.

#### Metadata

REGION (column A), BRAIN ID (column B) and NAME (column C) are reported as in Tab.1 BRAIN.

CATEGORY (column D) = It is specified that the site is off-site (not from archaeological excavation).

CONTEXTS (column E) = This is the general contexts of the site that was studied within interdisciplinary geo-stratigraphical investigation.

DESCRIPTIONS (column F) = This is the description of the point of sampling of plant macro or microremains; in many cases it was not possible to find this information, so many rows report ‘NA’ (Not Available).

NOTES (column G) = Additional information.

### TAB 4 BRAIN: Spot records with contexts and notes, in Italy

Botanical records can be isolated from very limited objects or contexts, reduced to a single plant accumulation, or residue in a container/pot or deposit on a body into a burial. While providing very circumscribed information, these analyses can be of high significance in interdisciplinary studies and provide very original point data. BRAIN includes 9 spot records.

#### Metadata

REGION (column A), BRAIN ID (column B) and NAME (column C) are reported as in Tab.1 BRAIN.

CATEGORY (column D) = This reports that these are ‘spot records’

TYPOLOGY (column E) = The typology of archeological site is attributed according to the archaeological literature, type of excavation and archeological study.

CONTEXTS (column F) = The type of contexts where the spot record was found.

DESCRIPTIONS (column G) = This is the description of the point of plant sampling within the site; in several cases this is not known for spot records (N/A).

NOTES (column H) = Additional information.

### TAB 5 BRAIN: List of sites (‘on-site’ and ‘off-site’ categories) with contexts, chrono-cultural phases and notes, in extra-Italy countries

A number of sites located outside Italy have been included in BRAIN, considering on-site (archaeological) sites included in syntheses of anthropogenic impact in the Mediterranean (EXI1-4); off-site sites of reference for anthropogenic impact in the Mediterranean (Lake Dorjan - EXI5), and on-site and off-site sites located in Malta, an island in the heart of the Mediterranean whose sites range from the Neolithic to the Bronze Age, thus in the phase of island’s cultural acme.

#### Metadata

REGION (column A), BRAIN ID (column B) and NAME (column C) are reported as in Tab.1 BRAIN.

AREA (column D) = This indicates that the sites are located in an area external to Italy, in Mediterranean countries.

COUNTRY (column E) = This is the geographical location of the site.

CATEGORY (column F) = It is specified whether these are ‘on-site’ or ‘off-site’ sites.

TYPOLOGY (column G) = Considering the ‘on-sites’, this is the typology of archaeological site, attributed according to the archaeological literature, type of excavation and archaeological study. In the other cases, it is repeated as typology that they are ‘off-sites’.

CONTEXTS (column H) = This is the general contexts of the site that was studied in archaeological studies (on-site) or within interdisciplinary geo-stratigraphical investigation (off-site).

DESCRIPTIONS (column I) = This is the description of the point of plant sampling within the site; in several cases this is not known (N/A).

CHRONO-CULTURAL PHASES (J) = In the case of on-sites, the main cultures and phases are reported as established in the archaeological literature, and mainly dated to prehistoric phases.

NOTES (column K) = Additional information.

### TAB 6 BRAIN: Type of botanical records from all sites in Italy and extra-Italy, with analyst teams and laboratories, and corresponding citations literature

**Fig. 6:**
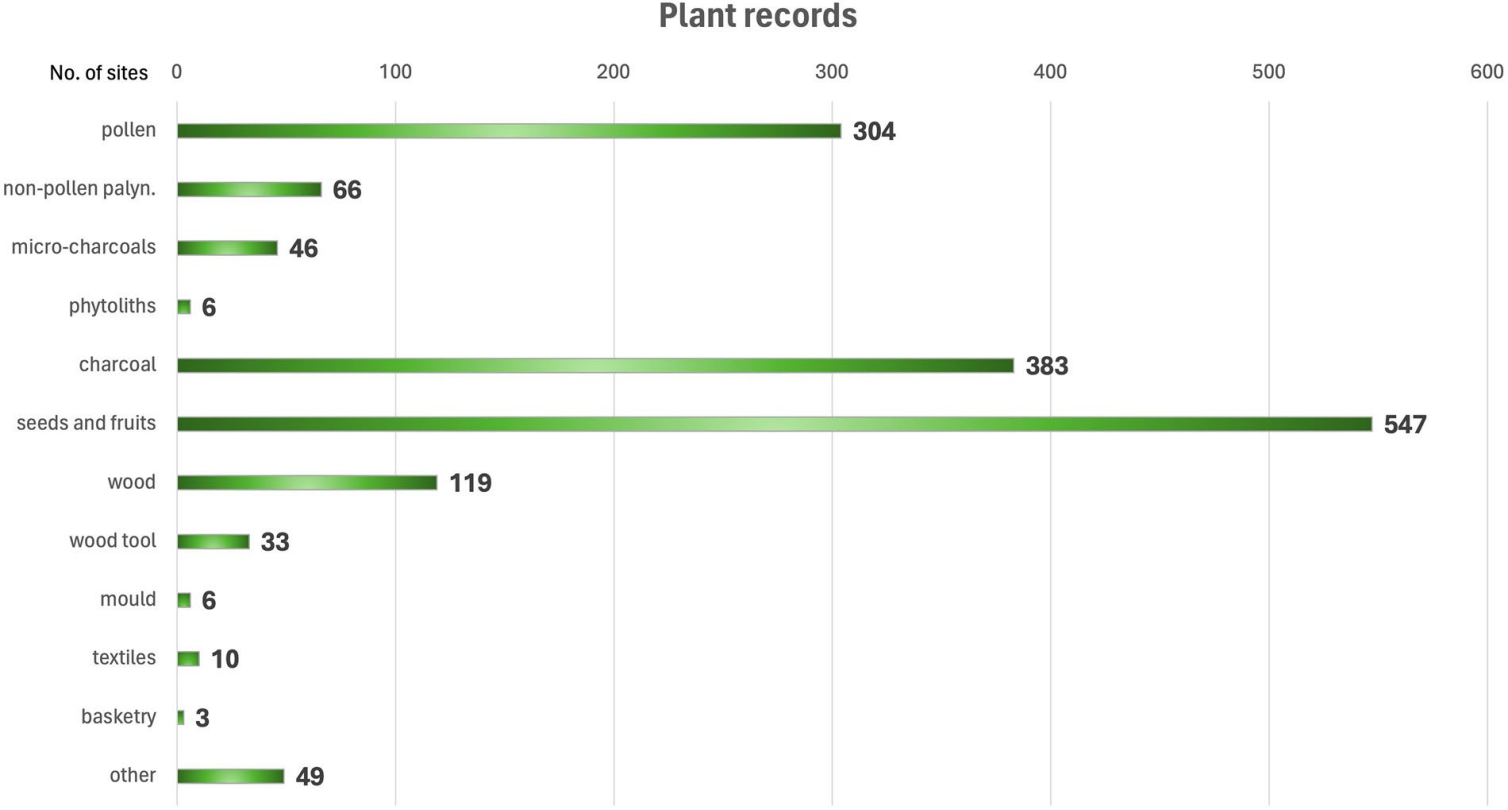
Total number of sites with archaeobotanical analyses in BRAIN, subdivided as main type of microscopical and macroscopical records, as resulting from the overall review of the literature on the subject.

Botany has brought an innovative biological perspective to archaeological contexts because outlined the role of plants as part of the ecosystem and not only a fragmented part as a function of human action (like the word ‘ecofact’ suggests). The list of plant remains that have been studied in one single site may be rich and complex, including pollen, seeds and fruits, woods or charcoals and also phytoliths, non-pollen palynomorphs, basketry and wood manufacts (Fig. 6). This means that the interpretation of the significance of their presence in the research context can be largely improved by comparing/integrating the different plant parts found during the analyses.

#### Metadata

REGION (column A), BRAIN ID (column B) and NAME (column C) are reported as in Tab.1 BRAIN.

PLANT RECORDS (column D) = This is the type of plant records studied in the site. One site can have one or many different types of plant records studied.

LABORATORIES (column E) = This is the team of researchers who made the analyses and the study of plant records. There may be a 1:1 correspondence between site and laboratory, but often it is several laboratories with different competences (e.g., on pollen and on plant macroremains) that have studied the same site.

REFERENCES (column F) = These are the citation(s) of the papers where archaeobotanical or palaeoecological data are published.

### TAB 7 BRAIN: List of references from which archaeobotanical and palynological analyses are available

The reference list includes the entire reference literature of the BRAIN database cited in Table 6 BRAIN. It represents an extensive inventory of 984 references from different sources (peerreviews scientific journals, monographies, book chapters, PhD theses, congress abstracts, grey literature) published since the 1980ies. The list of references is sorted by the alphabetic order of the first author and formatted by displaying: Author’s name(s), year of publication, title, source. Each row represents a reviewed reference from which has derived all the data merged under a single Site ID.

### TAB 8 BRAIN: Legends of all labels in the database

This table lists all the abbreviations used in the database about chrono-cultural phases, types of plant records, and the teams of researchers who made the analyses. Each abbreviation is accompanied by the related meaning.

#### Metadata

CHRONO-CULTURAL PHASES (columns A, B) = It shows the labels referring to the cultures and phases as established in the archaeological literature, and as reported in Tables 2 and 5. These abbreviations consist of the first letters (one or two) of the cultural phase (column A), and for each of them the meaning is reported (column B).

PLANT RECORDS (columns C, D) = The list of the labels used for the type of plant records – included in Table 6 – are reported (column C). The abbreviation consists of one or two initial letters of the botanical proxy; for plant macroremains, the first letter of the label is capitalized. Each abbreviation is provided with the related meaning (column D).

LABORATORIES (columns E, F) = The label of the affiliation of the teams included in the BRAIN network – shown in Table 6 BRAIN – are listed. The abbreviations consist of the acronym of the laboratory followed by the acronym of the Italian city or country for non-Italian teams (column E); for each of them the meaning is reported (column F). Independent researchers or laboratories not included in the BRAIN network are listed as Ot (others).

## Technical Validation

We presented the census of the sites that have been archaeobotanically studied across the Italian peninsula (including the Republic of San Marino) and the islands. We performed a thoroughly literature review to compile the dataset of Holocene archaeo/palaeo-botanical studies for assessing plant-cultural diversity in Italy and other Mediterranean regions using over 980 references from the 1980ies to present. The data quality of the dataset has been checked with the support of the BRAIN contributors and botanists of the Group of Palynology and Paleobotany of the Italian Society of Botany.

### Supplementary information

Dataset 1

Dataset 2

Dataset 3

Dataset 4

Dataset 5

Dataset 6

Dataset 7

Dataset 8

## Data Availability

No custom code was generated for this work.
